# Femtosecond Laser Micromachining of Soda–Lime Glass in Ambient Air and under Various Aqueous Solutions

**DOI:** 10.3390/mi10060354

**Published:** 2019-05-29

**Authors:** Lina Mačernytė, Julius Skruibis, Virgilijus Vaičaitis, Romualdas Sirutkaitis, Ona Balachninaitė

**Affiliations:** 1 Laser Research Center, Vilnius University, Saulėtekio ave 10, 10223 Vilnius, Lithuania; lina.macernyte0@gmail.com (L.M.); julius.skruibis@gmail.com (J.S.); virgilijus.vaicaitis@ff.vu.lt (V.V.); 2 Institute of Biochemistry, Vilnius University, Mokslininku str. 12A, 08662 Vilnius, Lithuania; romualdas.s@inbox.lt

**Keywords:** femtosecond laser micromachining, soda–lime glass, KOH solution, ultrashort pulses

## Abstract

We have studied femtosecond ablation of soda–lime glass sample under thin water film, under KOH and NaCl aqueous solutions films and their influence and benefits compared with ablation in the air atmosphere. These have been studied in case of the groove ablation using the infrared (IR) femtosecond laser. KOH aqueous solution film above the glass sample improved the ablation efficiency and led to the formation of the grooves with a higher aspect ratio when multi-scan glass cutting conditions were applied.

## 1. Introduction

Microprocessing of materials by ultrashort laser pulses is characterized by high quality, accuracy and efficiency. By using ultrashort laser pulses the heat-affected zone and the shock-affected zone can be reduced [1]. This feature is well adapted to high-precision microfabrication of materials and at the same time creation of various undesirable defects both on the surface of the material and in its volume is avoided [2,3,4]. In addition, ultrashort laser pulses have become a promising tool for processing transparent materials such as glass, fused silica, and sapphire. A focused ultrashort laser pulse can reach high enough intensity and cause nonlinear absorption effect in the transparent materials, allowing for performing microprocessing on the surface or inside of the materials [5,6,7,8,9,10].

To further improve the removal of the material during ablation, microprocessing may be carried out in a liquid medium. Due to laser radiation formed bubbles result in a liquid dynamics. This movement does not allow the material particles to settle down on the sample. In this way, the sediment is effectively removed from the formed structures. The additional liquid layer above the sample performs the function of cooling of the material as well and prevents the formation of the cracks in the sample [11,12]. The most commonly used liquid medium for microprocessing is water. An improvement of femtosecond laser ablation by using a water layer has been shown before at different materials, demonstrating a significant increase in ablation rates [13,14]. More and more experiments are carried out using aqueous solutions and non-water liquids. In this case, interaction with the laser radiation between the sample and the liquid begins to occur in chemical reactions that affect the speed and the quality of processing. Aqueous solutions of different concentrations of NaCl and water were used during laser induced plasma micromachining on aluminum specimen and it was shown that the salinity level of the solution determines the depth of the grooves formed in the material [15]. Microprocessing of silicon was performed in KOH aqueous solution (40% concentration) [16]. KOH solution is less toxic than the other chemicals used in silicon lithography. During the experiment, the laser beam was focused on a second surface of the silicon wafer, which was continuously moistened with KOH solution. The localized temperature increase caused even more corrosivity. Microprocessing of stainless steel was performed by immersing the specimen in different concentrations of NaCl aqueous solution [17]. The results obtained were compared to the processing performed on the dry surface of the sample. Obvious differences were observed in the quality and material removal rate when machining on a dry surface of the sample and immersing the sample in a NaCl aqueous solution.

In order to improve the production of coronary stents used in medicine, femtosecond laser microprocessing of nitinol (nickel and titanium alloy) was performed in water [18]. The results showed the amount of debris, sediment and dust to be significantly lower in the case of processing with a water layer above the sample. At laser-induced plasma temperatures and due to UV radiation, molecules in liquids that are neutral at room temperature may be excited, ionized or dissociated and thereby become chemically active [19]. Optical emission spectra were used to investigate the chemical nature of products of graphite ablation in water, benzene, n-hexane and carbon tetrachloride [20]. In many articles, the formation of oxides on the target surface [21] or in plasma [20] has been observed at laser ablation of solids in water, which indicates that water dissociates and provides oxygen for chemical reactions. The catalytic activity of solid surfaces changes due to laser etching in water as well as described above. Therefore, advantages of liquid-assisted processing is attracting much attention in laser micromachining applications.

In this article, a femtosecond 1030 nm wavelength laser radiation was used to perform microprocessing of soda–lime glass in ambient air, in water-assisted environment, under KOH and under NaCl aqueous solutions. The quality, the depth and the width of the grooves formed by femtosecond laser microprocessing on the surface of the soda–lime glass sample were investigated.

## 2. Experimental Setup

**Figure 1:**
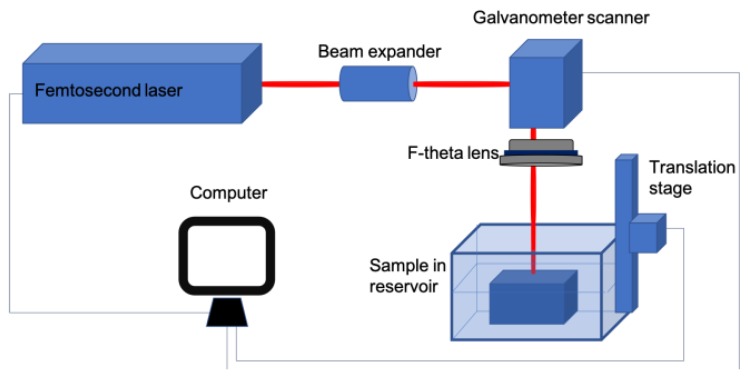
Experimental setup of a femtosecond laser processing system.

The experiments were performed using the Carbide® Yb:KGW femtosecond laser system (Light Conversion Ltd., Vilnius, Lithuania) operating at 1030 nm (pulse duration 280 fs, average power up to 5 W and repetition rate 60 kHz). The maximum laser energy of a single femtosecond pulse was approximately 80μJ. The experimental scheme is presented in Figure 1. Laser beam passing through the beam expander is directed to the two axis galvanometric scanner (ScanLab Inc., Munich, Germany), controlled by SCA fabrication software (Altechna Ltd., Vilnius, Lithuania), which was used for fast and precise positioning of mirrors to deflect laser beams.

After the laser beam passed through the scanner, it was focused by a F-Theta lens (f = 75 mm) in the normal incidence onto the sample (focal spot diameter 20 μm), which was mounted on a three-dimensional motorized positioning stage (Standa Inc., Vilnius, Lithuania) for precise positioning of the sample. Galvanometric scanners in combination with F-theta lenses can produce large scanning fields and scanning rates of the order of m/s or greater. Micromachining was carried out on soda–lime glass of 1-millimeter thickness (Thermo Scientific Inc., Waltham, MA, USA). Chemical composition of the glass sample: SiO_2_ 72.20%; MgO 4.30%; Na_2_O 14.30%; Al_2_O_3_ 1.20%; K_2_O 1.20%; Fe_2_O_3_ 0.03%; CaO 6.40%; SO_3_ 0.30%. The sample was processed under a thin (~0.6 mm thick) liquid layer and without a liquid layer. The thickness of a particular liquid layer was chosen based on our previous experiments on glass cutting with high repetition-rate femtosecond laser pulses using a similar experimental setup [22]. The desired thickness of the layer was formed by submerging the glass sample in a reservoir. An optical microscope BX51 (Olympus, Tokyo, Japan), profilometer Sensofar PLμ2300 and an electronic microscope TM-1000 (Hitachi Tabletop, Tokyo, Japan) were used to analyze the depth and the quality of the formed grooves.

## 3. Results and Discussion

### 3.1. Preparation of Specimens

Soda–lime glass samples were used for laser micro-processing. Typical dimensions of the glass sample was 75 × 25 × 1 mm. The specimens were placed in a special reservoir in which the sample was processed both in liquid medium and in air. Laser treatment was performed on a dry surface of the glass and when the glass sample was immersed in water, KOH and NaCl aqueous solutions. The NaCl was dissolved in distilled water at room temperature. Ten solutions with different concentrations of NaCl/H_2_O (from 1 g/100 mL to 10 g/100 mL) were prepared. The influence of KOH aqueous solution on micro-processing was also analyzed. The concentration of KOH solution ranged from 1% to 35%. In each experiment, the thickness of liquid layer over the sample was attempted to maintain stable and equal to about 0.6 mm. During the experiment, various parameters that could affect the depth and quality of the formed grooves were changed. As a scanning algorithm was chosen a raster scanning [23]. The beam was scanned in straight lines: 7 mm in the positive *y*-axis direction, then 0.3 mm in the positive *x*-axis direction, and 7 mm in the negative *y*-axis direction, and further 0.3 mm in the positive *x*-axis direction. This path was repeated several times.

### 3.2. Evaluation of the Groove Depths

**Figure 2:**
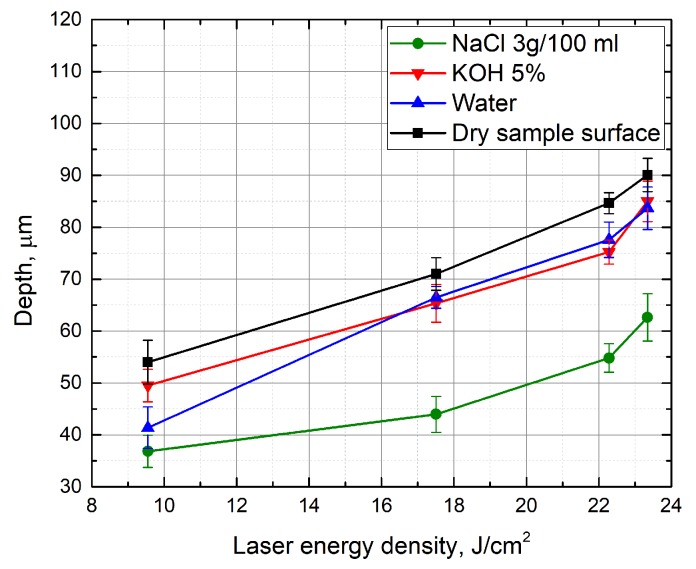
Depth of the grooves formed in the glass sample in dependence on the laser energy density. Grooves were formed while the glass sample surface was dry and immersed in different environments—5% KOH, 3 g/100 mL NaCl solutions, water. Scanning speed—100 mm/s, number of scans—15, pulse repetition rate—60 kHz.

**Figure 3:**
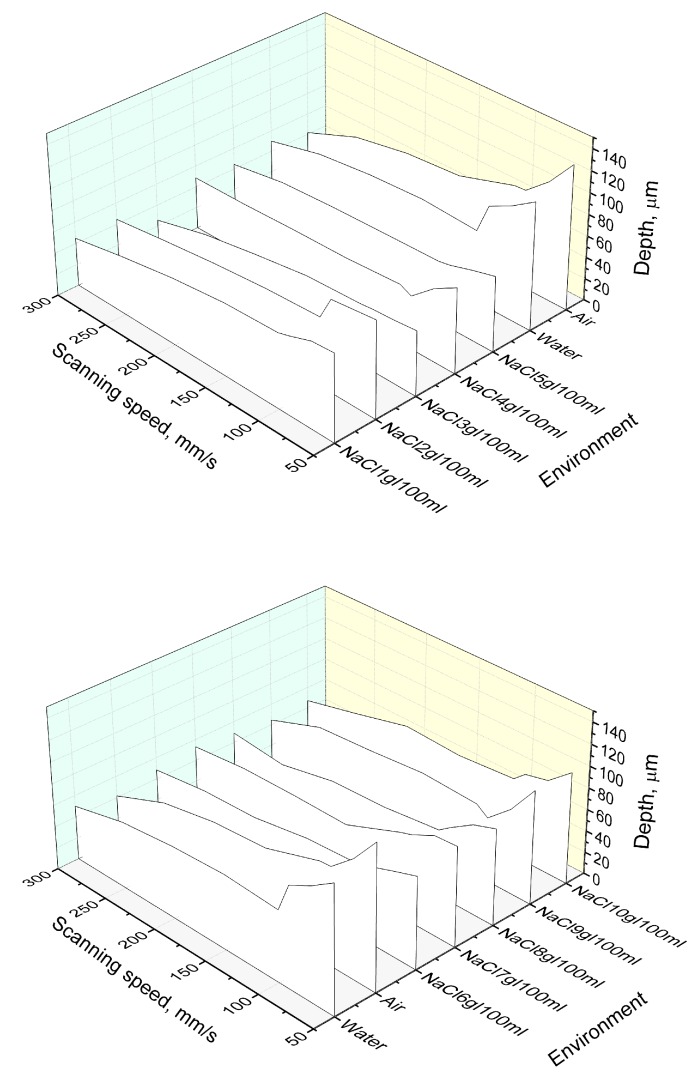
Depth of the grooves formed in the glass sample in dependence on the scanning speed. Grooves were formed while the glass sample surface was dry and immersed in different concentrations (from 1 g /100 mL—to—10 g/100 mL) of NaCl aqueous solutions, and water. Number of scans—15, average power—5 W, pulse repetition rate—60 kHz.

**Figure 4:**
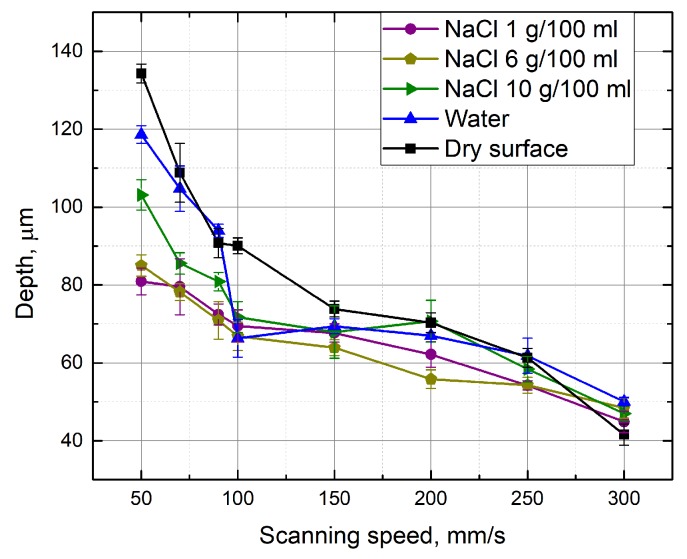
Depth of the grooves formed in the glass sample in dependence on the scanning speed. Grooves were formed while the glass sample surface was dry and immersed in 1 g/100 mL, 6 g/100 mL and 10 g/100 mL concentrations of NaCl aqueous solutions, and water. Number of scans—15, average power—5 W, and pulse repetition rate—60 kHz.

**Figure 5:**
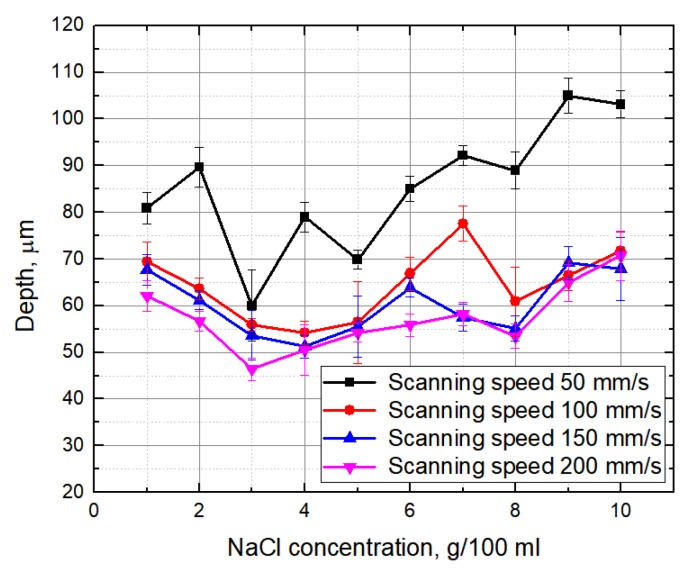
Depth of the grooves formed in the glass sample in dependence on the concentration of NaCl aqueous solution. Measurements were repeated at constant scan speeds: 50 mm/s, 100 mm/s, 150 mm/s, 200 mm/s. Number of scans—15, average power—5 W, pulse repetition rate—60 kHz.

Firstly, the dependence of the groove depth on laser energy density was evaluated (Figure 2). Measurements were made when the laser beam scanning speed (100 mm/s) and the number of scans (15 scans) were kept constant. It can be seen that, independently of the environment in which the glass plate is processed, the depth of the formed grooves is nearly linearly dependent on laser energy density. Figure 3 and Figure 4 show the dependence of the formed grooves depths on the scanning speed when the sample is processed in different media—in NaCl aqueous solution, water and on a dry surface of the glass. All measurements were performed at a fixed number of scans (15), average laser power (5 W), and pulse repetition rate (60 kHz). The concentration of NaCl aqueous solution was changed from 1 g/100 mL to 10 g/100 mL. It can be seen that, in all cases, with increasing scanning speed, the depth of grooves decreases. At a low scanning speed, the number of pulses per unit area is higher. For this reason, the ablation process takes longer, resulting in more effective ablation. It is also noticed that deeper grooves are formed when there is no additional water or NaCl aqueous solution layer above the specimen. However, increasing the concentration of the NaCl aqueous solution reveals a tendency towards increasing the depth of the grooves, which is particularly evident at a low scanning speed (50 mm/s) (Figure 5). This dependence is probably due to the fact that chlorine acts as a catalyst for the chemical reactions using NaCl solutions in laser microprocessing [19].

**Figure 6:**
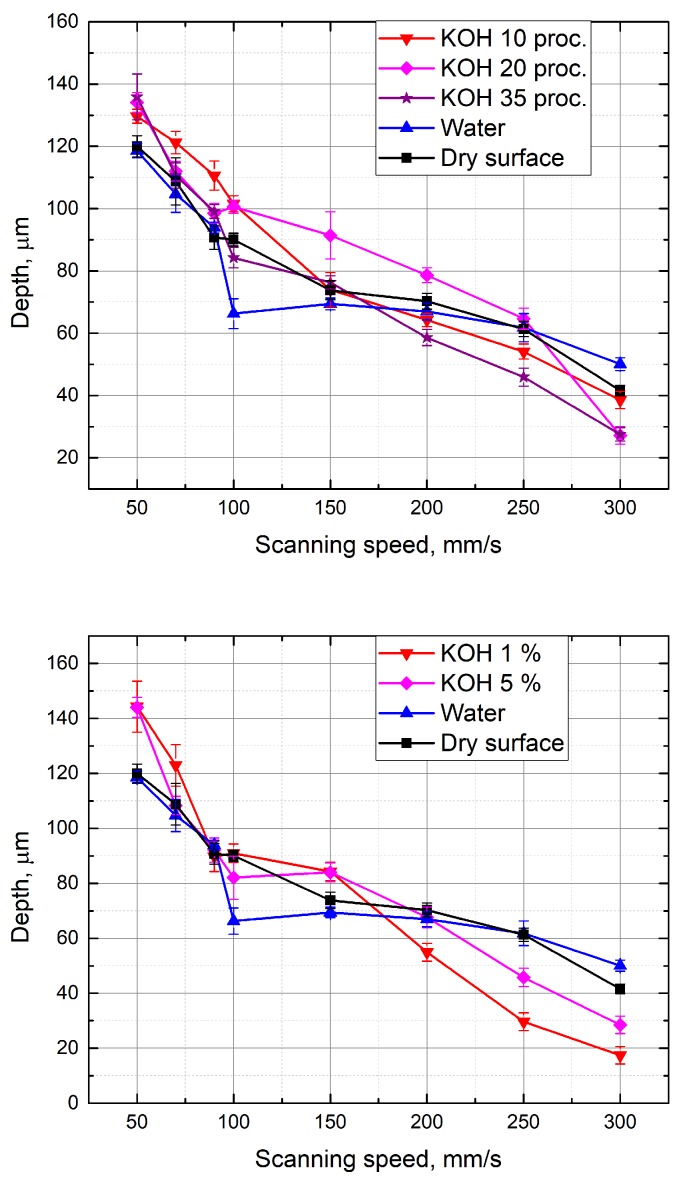
Depth of the grooves formed in the glass sample in dependence on the scanning speed. Grooves were formed while the glass sample surface was dry and immersed in different concentrations (1%, 5%, 10%, 20%, 35%) of KOH aqueous solutions, and water. Number of scans—15, average power—5 W, pulse repetition rate—60 kHz.

**Figure 7:**
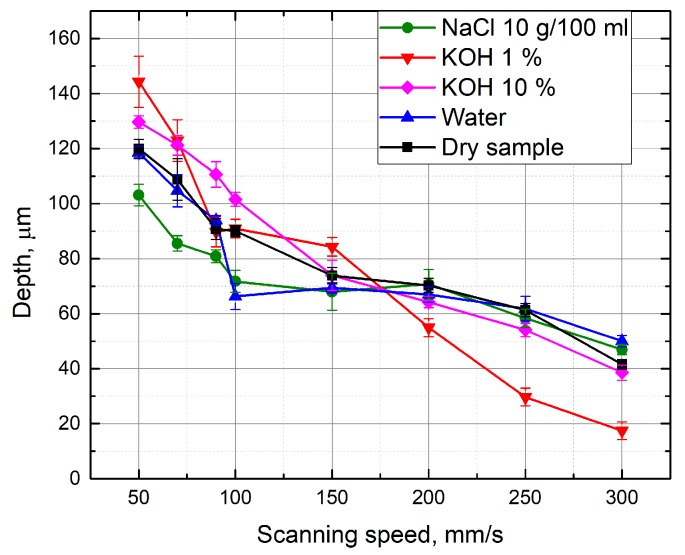
Depth of the grooves formed in the glass sample in dependence on the scanning speed. Grooves were formed while the glass sample surface was dry and immersed in different concentrations (1%, 10% KOH, 10 g/100 mL NaCl) aqueous solutions, water. Number of scans—15, average power—5 W, pulse repetition rate—60 kHz.

**Figure 8:**
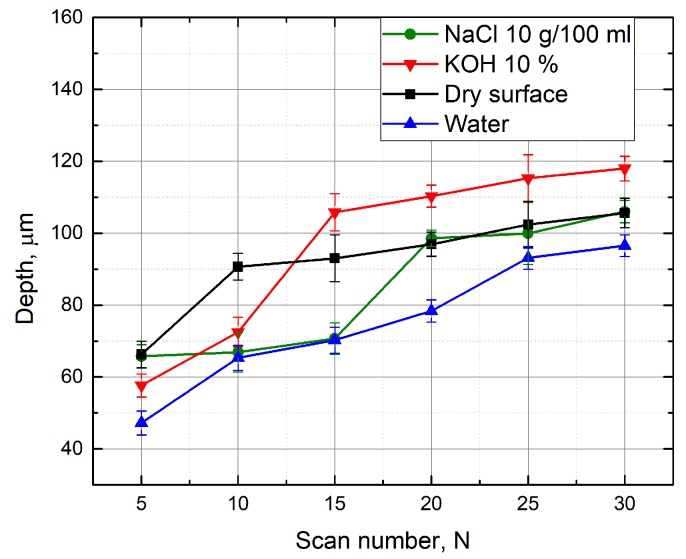
Depth of the grooves formed in the glass sample in dependence on the scan number. Grooves were formed while the sample surface was dry and immersed in different liquids—10% KOH, 10 g/100 mL NaCl aqueous solutions, and water. Scanning speed—100 mm/s, number of scans—15, average power—5 W, pulse repetition rate—60 kHz.

A similar experiment was performed with KOH aqueous solutions above the glass sample. Measurements were made at different concentrations of KOH (from 1% to 35%). The depth of the formed grooves linearly depends on the scanning speed (Figure 6). Using KOH aqueous solutions at different concentrations, the grooves are deeper than the grooves formed with the water layer above the specimen at low scanning speed. When KOH concentration is 1% and 5%, the grooves are about 8% deeper than the grooves obtained by working on a dry surface of the sample (at 50 mm/s scanning speed). Deeper grooves are also formed by immersing the sample in a 10% KOH solution, and when the scanning speed is changed from 70 mm/s to 150 mm/s. By selecting a scan speed from 100 mm/s to 250 mm/s and processing the material in a 20% KOH solution, 8% deeper grooves (in comparison to grooves formed on the dry sample) were also obtained. The increase in groove depths was observed possibly because the laser radiation causes a local increase in temperature, which increases the corrosion properties of the KOH solution. For this reason, more material is removed during the ablation [10]. It is also noticed that the depth of the grooves formed in KOH aqueous solution is clearly higher than that when the sample was under a NaCl solution. Figure 7 shows a comparison when processing is performed in different media. Each measurement maintains a constant number of scans (15), average laser power (5 W), and pulse repetition rate (60 kHz). When the scanning speed is equal to 50 mm/s, the formed grooves in the 1% KOH solution are about 1.4 times deeper than the grooves obtained by immersing the specimen into a NaCl aqueous solution at a concentration of 10 g/100 mL. Deeper grooves are also observed at a 50 mm/s–150 mm/s scanning speed range, while immersing the sample into a 10% KOH solution during processing. This can be explained by knowing that the KOH solution has stronger corrosion properties. However, as shown in Figure 7, with a high scan speed (200–300 mm/s), the depths of grooves formed in solution of NaCl (10 g/100 mL) are deeper than the grooves obtained at a 1% KOH solution layer above the sample. Figure 8 shows the depth of the formed grooves dependent on the scan number by micro-processing in different media—on a dry surface of the sample, under water and under NaCl (10 g/100 mL) and KOH (10%) aqueous solutions. As the number of scans increases, the depth of the grooves increases, and with each pass increases the amount of material removed. However, at the first 10 scans, the depths of the grooves formed when the sample was under 10% KOH aqueous solution is less than the depths of the groove when the sample surface was dry. It takes time for corrosivity of KOH aqueous solution to take place. In addition, after 15 or more scans, the grooves formed while the sample was under 10% KOH aqueous solution were the deepest ( 9% deeper than for dry surface of the sample).

### 3.3. Evaluation of the Depth-To-Width Ratio of the Formed Grooves

**Figure 9:**
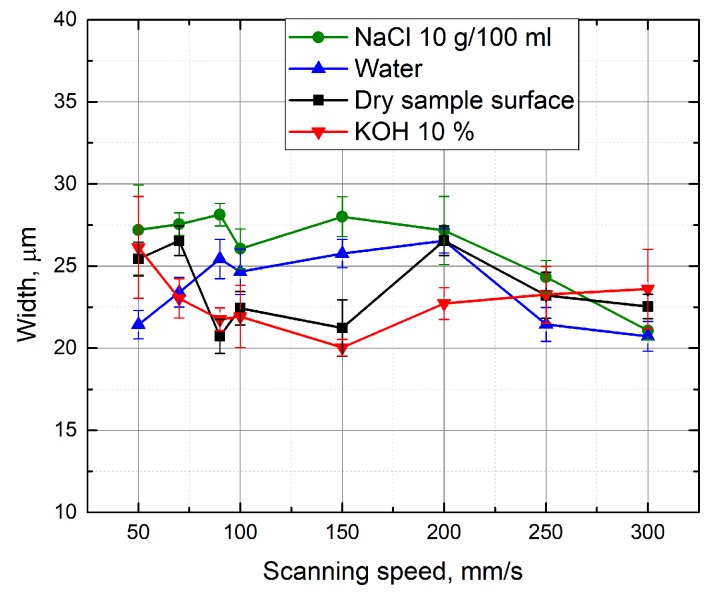
Width of the grooves formed in the glass sample in dependence on the scanning speed. Grooves were formed while the glass sample surface was dry and immersed in different liquids—10% KOH, 10 g/100 mL NaCl solutions, and water. Number of scans—15, average power—5 W, pulse repetition rate—60 kHz.

**Figure 10:**
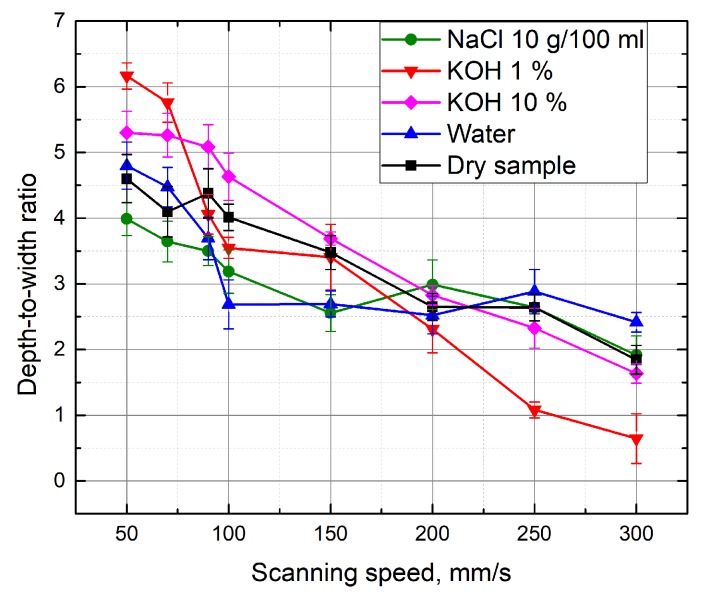
Depth-to-width ratio of the grooves formed in different media depending on the scanning speed.

Figure 9 shows the width of the formed grooves in dependence to the scanning speed. By performing the micro-machining in different media, the width of the formed groove varied from 20 μm to 28 μm. In order to determine the machining efficiency, the depth-to-width ratio of the grooves was evaluated. Figure 10 shows the depth-to-width ratio of the grooves formed in different media depending on the scanning speed. It can be seen that the highest values of this parameter are available at a low scanning speed (50 mm/s). Dependence is valid for processing in various media. In this case, the maximum groove depth and width ratio is obtained when the grooves are formed with an additional 1% KOH solution layer above the sample.

### 3.4. Evaluation of Grooves Quality

**Figure 11:**
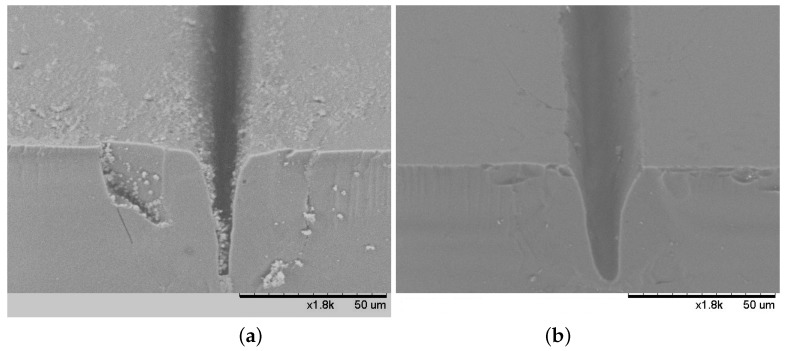
SEM photographs of the grooves formed while the glass sample surface was dry (**a**) and immersed in KOH aqueous solution (**b**).

The quality of the grooves formed in the glass sample was evaluated using electronic and optical microscopes. Figure 11 shows that a better groove quality is monitored by laser micro-processing on a dry surface of the sample than in KOH aqueous solution. In this groove formed in the liquid, some damage to the surrounding surface is observed and a higher groove contamination. Similar defects are detected in all specimens that were immersed in various liquids during the experiment. The deteriorated quality could have been affected by the changing thickness of the liquid above the surface to be treated. Prolonged evaporation caused by laser radiation prevented the maintenance of constant liquid content. However, when processing in liquids, there is no observed sediment accumulation at the edges of the grooves (Figure 11).

**Figure 12:**
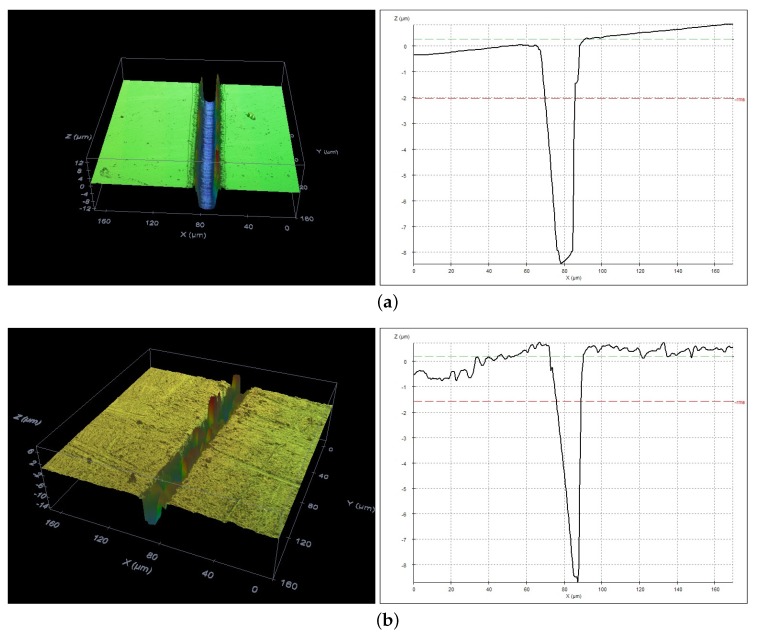
Profiles of the ablated grooves of soda–lime glass in 20% KOH aqueous solution (**a**) and in air (**b**).

Profiles of the formed grooves were evaluated by an optical profilometer (Figure 12). It was found that the grooves formed by laser irradiation in air and in liquid are quite different. Narrow conical “V”-shape grooves were observed in case of ablation in air, while flat bottom “U”-shape grooves were produced in liquids. The reason for that could be the transformation of the Gaussian intensity profile of the laser beam during the propagation through the liquid.

### 3.5. Comparison of the Presented Techniques with Other Studies

Most of the investigations of the microfabrication process under KOH and NaCl aqueous solutions were performed on metals, silicon carbide, silicon and ceramic materials [24,25,26]. In some of the studies, it was shown that the application of a chemically active KOH solution during silicon microfabrication does not necessarily help to increase the etching rate and even it can stop the etching of Si surface [27]. During liquid assisted femtosecond laser machining of quartz plates in KOH solution, etching enhancement along E-field of a linearly polarized laser beam was observed [28]. To our knowledge, no studies were performed on femtosecond laser micromachining of soda–lime glass under KOH or NaCl aqueous solutions. Other interesting results were presented on the microfabrication in organic solutions, e.g., methanol, acetone, ethanol and toluene [29,30]. Alcohol-assisted femtosecond photoetching of silicon carbide was shown to be beneficial to decreasing the redeposition of debris and increasing the ablation depth [31]. In order to compare our results in aqueous solutions with femtosecond laser micromachining of soda–lime glass in organic solutions, additional experiments must be done.

## 4. Conclusions

We have experimentally investigated the multiple scans groove formation in soda–lime glass samples with 280 fs duration laser pulses. Experiments were conducted in different environments: in ambient air, under water, under KOH aqueous solution and under NaCl aqueous solutions. By applying NaCl aqueous solution film on the glass sample and increasing NaCl aqueous solution concentration up to 10 g/100 mL shows a tendency for deepening of the grooves, which is especially evident at a low scan speed (50 mm/s). The possible reason for this is that, after dissolving NaCl in distilled water, due to the Na+ and Cl- ions contained in the solution the energy of ionization of the medium is reduced, and, at the same time, the threshold plasma intensity is reduced, resulting in an increase in the density of the charge in the area of damage. Ions are also likely to accelerate the process of multi-photon ionisation and initiate an avalanche ionization. Another explanation for this dependence is also possible. When a certain concentration of NaCl solution is reached, chlorine ions can be involved in chemical reactions at high temperatures, leading to the disintegration of the substance. However, by applying NaCl aqueous solution film on the glass sample, the depths of the formed grooves at the same conditions were lower in comparison to the grooves formed on the dry sample surface or under water and KOH aqueous solution. Investigation showed that the applied KOH aqueous solution film improved the ablation efficiency when multi-scan glass cutting conditions were applied. Using KOH solutions of different concentrations led to the groove’s depth being greater than the grooves that were formed in the water layer above the sample and by using a small scan speed (50 mm/s). Deeper grooves are also formed when processing in 10% KOH and 20% KOH aqueous solutions. In this case, the range of the scanning speeds are equal to—70 mm/s–150 mm/s and 100 mm/s–250 mm/s (at average laser power—5 W, pulse repetition rate—60 kHz), respectively. The increase in the groove depths is observed because the laser radiation causes a local increase in temperature, which increases the rate of corrosion of the KOH solution. In addition, the formation of deeper grooves may have been influenced by porosity of the material, which increases the area of interaction. After laser micro-processing of glass samples in various environments—in different concentrations of NaCl, KOH aqueous solutions, in water and on a dry surface of the sample—the ratio of the formed grooves depth-to-width was evaluated. The greatest value of this parameter is obtained when the sample is processed in a 1% KOH solution at a scanning speed of 50 mm/s. Meanwhile, when the scanning speed is 100 mm/s, the groove depth-to-width ratio is the best at 10% KOH. A higher number of pulses per unit area at a lower scan speed results in a higher temperature, at which both lower concentrations of KOH and immersion of glass samples yield comparable groove depth-to-width ratios, such as grooves, formed when the specimen was immersed into a higher concentration KOH solution at a higher scan speed. However, better quality of the formed grooves is observed by laser micro-processing on a dry surface of the sample. This was probably due to changing liquid thickness above the working surface and due to high thermal expansion coefficient of soda–lime glass. Possibly the cracks could be avoided in the glasses with lower thermal expansion coefficients. Future investigations will be needed to clarify the crack formation mechanism. However, under liquid films, grooves formed were free from redeposited debris. Our findings suggest that, under certain conditions, KOH-assisted ablation could be effective in laser processing of glass applications.
